# High-Resolution Ultrasound and Magnetic Resonance Imaging of Abnormal Ligaments in Thoracic Outlet Syndrome in a Series of 16 Cases

**DOI:** 10.3389/fnins.2021.817337

**Published:** 2022-02-02

**Authors:** Suren Jengojan, Maria Bernathova, Thomas Moritz, Gerd Bodner, Philipp Sorgo, Gregor Kasprian

**Affiliations:** ^1^Department of Biomedical Imaging and Image-guided Therapy, Medical University of Vienna, Vienna, Austria; ^2^Radiology Department, Christchurch Hospital, Christchurch, New Zealand; ^3^Neuromuscular Imaging Center Döbling, Vienna, Austria; ^4^Karl Landsteiner Private University, Krems, Austria

**Keywords:** thoracic outlet syndrome, cervical ribs, fibrous ligaments, high-resolution ultrasound, magnetic resonance imaging, fiber tracking

## Abstract

**Introduction:**

Neurogenic thoracic outlet syndrome (NTOS) is a complex entity that comprises various clinical presentations, which are all believed to result from mechanical stress to the brachial plexus. Causes for the stress can include fibrous bands, spanning from the transverse processes, stump, or cervical ribs to the pleural cupula. The aim of this case series is to document how the combined potential of high-resolution neurography, including high-resolution ultrasound (HRUS), and magnetic resonance imaging (MRI) can be used to identify, anatomical compression sites, such as stump ribs and their NTOS associated ligamentous bands.

**Materials and Methods:**

Retrospective chart and image reviews identified patients, who underwent HRUS between 2011 and 2021 and the diagnosis of NTOS caused by accessory ligaments was subsequently confirmed by radiological imaging (MRI) and/or surgical exploration.

**Results:**

Sixteen patients were included in this study. In all cases, a ligament extending from the tip of a stump rib to the pleural cupula could be depicted. In all cases, these structures led to compression of the lower trunk of the brachial plexus. All surgically explored cases confirmed the radiological findings.

**Conclusion:**

This case-series demonstrates that HRUS and MRI can directly and reliably visualize accessory costocupular ligaments and a stump rib in patients with symptoms of NTOS. HRUS may be used as the first imaging modality to diagnose suspected NTOS.

## Introduction

The thoracic outlet syndrome (TOS) is a complex syndrome that comprises various clinical neurovascular entities, which are all believed to result from mechanical stress in the region that is clinically referred to as the thoracic outlet region (Ferrante, 2012). The anatomical complexity of this region has been described as long ago as the end of the 19th century and still poses a considerable challenge to clinicians and radiologists (Zuckerkandl, 1876; Martinoli et al., 2010).

Pathologic processes or predispositions leading to a TOS can be divided into anatomical or functional causes (e.g., postural and respiratory alterations) (Vanti et al., 2007). Depending on the structures affected, TOS is divided into an arterial type (A-TOS), a venous form (V-TOS), and into the most common, neurogenic TOS (NTOS), which represents approximately 90% of TOS. NTOS is further divided into the so-called “true”-NTOS, where there are objective anatomical and/or electrodiagnostic findings and the so-called “disputed”-NTOS where such findings cannot be established. The percentage of disputed NTOS has been reported at a rate of as much as 85% in the literature (Vanti et al., 2007). A potential cause of NTOS is compression of the brachial plexus trunks within the scalene musculature (Leonhard et al., 2016).

The clinical presentation of TOS varies substantially and, in addition to vascular symptoms, most commonly involves wasting of the thenar and hypothenar eminence, of the ulnar intrinsic muscles, and the medial forearm muscles (Baumer et al., 2014). The diagnosis of TOS is frequently established by clinical examination, with false-positive rates of typical clinical tests ranging from 9 to 77% (Nord et al., 2008). Previously, the task of imaging in clinically suspected TOS was mainly limited to detecting osseous anomalies and angiographic or color Doppler flow changes in the subclavian vessels during postural changes (Braun, 2010).

Compression and hemodynamically relevant stenosis of the subclavian artery occurs due to cervical ribs, scalenus anticus muscle, costoclavicular narrowing.

In up to 85 % of the A-TOS cases cervical ribs are present resulting in A-TOS with vascular and neurological symptoms.

Compression of the subclavian artery leads to upper limb ischemia and cooling, pain, fatigability, claudication, pallor, and decrease or absence of distal pulsations, while neurological symptoms are only secondary to the vascular anomalies in A-TOS (Ferrante and Ferrante, 2017).

**FIGURE 1:**
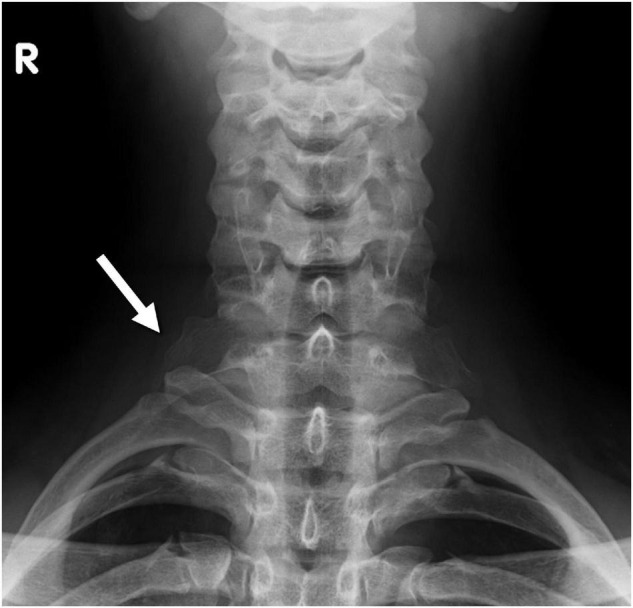
Radiograph of the cervical spine shows a stump rib on both sides (white arrow).

However, while only 5–10% of osseous anomalies are considered causative of TOS, up to 66% of NTOS patients show soft tissue variants at surgical exploration, such as muscle or ligament variants that affect the neurovascular bundle (Makhoul and Machleder, 1992; Roos, 1999; Figure 1).

**FIGURE 2:**
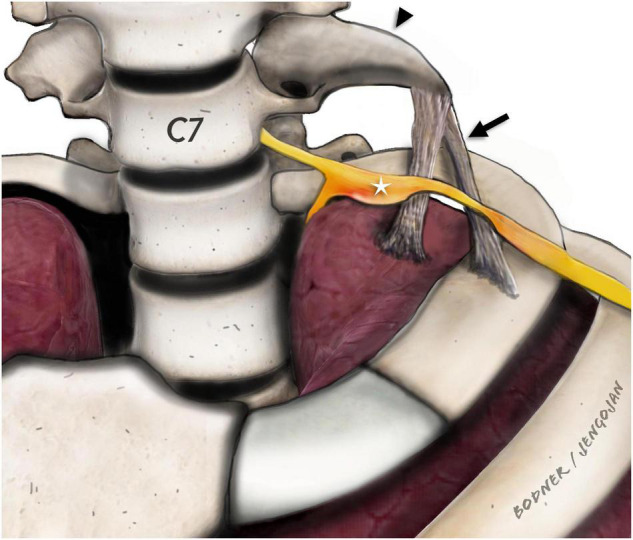
Para-sagittal/para-coronal illustration of the stump rib (black arrowhead) and the fibrous ligaments (black arrow). Adjacent to the tip of the stump rib (C7), the fibrous ligaments are drawn which extend either to the first rib or to the pleural cupula as described by Emil Zuckerkandl. The inferior brachial plexus (C8 and T1) components (white star) are displaced and enlarged with obvious constriction at the direct level of the crossing fibrous ligaments. In our study, we only detected ligaments extending to the pleural cupula.

Among the soft tissue variants, the ligaments in the thoracic outlet region have been a particular focus of attention in patients with NTOS. Juvonen et al. (1995) have reported variable fibrous cervical bands in the thoracic outlet region in more than 50% of the general population and in more than 90% of patients treated surgically for NTOS (Urschel and Grewal, 1995). Roos et al. have described several different variants of such fibrous bands. Two distinct types (Roos Types I and II) run from the tip of a cervical rib or hypertrophic transverse process to the upper aspect of the first rib and partly to the pleural cupula. The course of these ligaments typically crosses the C7 and/or C8 cervical root and the inferior trunk of the brachial plexus and may be a cause of entrapment of these structures (Roos, 1976; Figure 2).

According to the literature there is, no well-defined or precise naming of rudimentary/incomplete cervical ribs, we therefore defined a pointed, elongated C7 transverse process or incomplete cervical rib with a fibrous ligament arising at its tip as stump rib throughout this manuscript.

Until today these ligaments have been hardly approached by imaging. The emergence of more sophisticated magnetic resonance imaging (MRI) and high-resolution ultrasound (HRUS) has changed the technical capabilities and the possibilities to visualize these small structures in an anatomical region, which is generally difficult to be accessed. As yet, there are only very few studies that have focused on the imaging of such ligaments and only one case report involving HRUS has been published (Simon et al., 2013; Baumer et al., 2014).

The use of clinical high field (3 tesla) MR has led to improvements in spatial resolution and better signal-to-noise ratios in visualizing small structures such as peripheral nerves, including the difficult system of the brachial plexus (Mallouhi et al., 2012). Diffusion tensor imaging (DTI) has shown promising results in the assessment of the brachial plexus in peripheral nerve entrapment syndromes (Makhoul and Machleder, 1992; Roos, 1999; Braun, 2010; Vargas et al., 2010; Tagliafico et al., 2011; Ferrante, 2012). Entrapment of the brachial plexus produced by structures, such as cervical ribs and fibrous bands, has been reported in seven of thirty patients with clinical signs of TOS using 3 Tesla MR-Neurography with a positive predictive value of 100% (Baumer et al., 2014). To our knowledge, the combined use of HRUS and MRI with and without the use of peripheral nerve tractography has not been systematically evaluated in a series of patients with anatomical compression of the brachial plexus.

The aim of this retrospective multimodal imaging study is to: (a) demonstrate the reliable possibility of visualization of ligaments in the thoracic outlet region by means of HRUS; (b) review the HRUS features of the ligaments and compression; and (c) correlate these features to the clinical, surgical, and MRI findings. Furthermore, we seek to evaluate whether the deterministic DTI based tractography of the brachial plexus can provide additional imaging aspects in anatomical conditions that lead to NTOS.

## Materials and Methods

The study was conducted according to the Declaration of Helsinki and was approved by the local Institutional Ethics Review Board (IRB) of the Medical University of Vienna.

A retrospective analysis was conducted of the HRUS images of all patients who presented with a final diagnosis of NTOS caused by abnormal ligaments who had been treated surgically or who had had a 3 Tesla MRI examination. Ultrasound examinations were performed using HRUS equipment (GE Logiq e9, ML6-15-D, and L8-18i-D). Findings were documented in the para-axial, para-coronal, and para-sagittal planes using both still images and dynamic video sequences in both the resting position and in dynamic arm movement and forced inspiration and expiration.

### High-Resolution Ultrasound Image Evaluation

The HRUS examinations were reviewed in consensus by two radiologists with more than 5 years of experience in peripheral nerve imaging. Images were assessed for an increase in nerve diameter defined as a focal or regional increase of the cross-sectional area (CSA) by more than 25% or signs of fascicular swelling, as well as a significant course deviation, defined as a sudden pointed kink of the whole nerve with a sustained change in the course by more than 30 degrees (minimal value in all imaged planes). Furthermore, the images were evaluated with regard to hyperechoic linear structures that resembled ligaments, their respective courses, and possible subclavian artery narrowing.

### Magnetic Resonance Imaging and Diffusion Tensor Imaging Evaluation

Existing MRI examinations of those who matched the inclusion criteria and were retrospectively assessed.

The MRI examinations [coil: 16-channel neurovascular coil; imaging sequences: sagittal and transversal T2-weighted (T2w) turbo spin echo (TSE), parasagittal short term inversion recovery (STIR), paracoronal T1w TSE, parasagittal T2W volume isotropic turbo spin-echo acquisition (VISTA), axial DTI] were reviewed in consensus by two radiologists with more than 5 years’ experience in peripheral nerve MR imaging. Nerve diameter enlargement, increased T2W signal, as well as significant nerve fiber course deviation was assessed. In addition, the images were evaluated with regard to the fibrous bands described on HRUS, which affected the brachial plexus or subclavian vessels.

The DTI source data (axial, echo-planar, single-shot DTI sequence, *b*-values of 0 and 700 s/mm2, 16 diffusion encoding directions, slice thickness 3 mm, acquisition time 6 min 10 s) was post-processed using the Philips Intellispace Workstation (Philips Medical Systems, Best, Netherlands). The transaxial DTI data was used to identify the nerves and myelon. Deterministic tractography of the brachial plexus was performed one experienced examiner by placing regions of interest (ROIs) along the course of the respective nerves, using standard fiber reconstruction thresholds (*FA* = 0.15, angle change = 27°) and a FACT (fiber assignment by continuous tracking) algorithm. The post-processing procedure took around 20–30 min per plexus.

### Clinical Record Review

The patient history was accessed in the local RIS by searching for documents relevant for the study of the central clinical problem (NTOS). Radiological reports, reports of functional tests (e.g., nerve conduction studies), surgical reports, and discharge letters were analyzed for information relevant to the study. Surgical image documentation was also accessed, if applicable.

## Results

There were 158 patients evaluated using HRUS for suspected NTOS during the study period. Of those, 16 patients were diagnosed with NTOS, and were, therefore, eligible for the study [mean age 47 years (SD, 15 years), 13 females, 3 males]. All of them reported spontaneous development of clinical symptoms that showed a broad spectrum from diffuse pain radiating from the shoulder down the upper extremity, to distinct atrophy of the C8 muscles (for details, see Table 1). The mean time to final diagnosis was 8 years (SD, 5 years) based on the responses of 11 patients. In five patients, the duration of symptoms could not be reliably determined.

**TABLE 1:** Detailed chart review of all patients included in this study. In the top column the symptoms, results, therapies and examinations considered in this study are listed.

Case number/ Age/Sex	Neurologic symptoms	Muscular/Sensory (M, S) symptoms	Affected side	Symptom duration	Previous surgery	Sonography	MR (3 Tesla)	Treatment
1/29y/F	Median nerve atrophy intrinsic muscles	M, S	bilateral	15 years	Unilateral (left) first rib and subsequent cervical rib resection	Stumpl rib C7, accessory ligament from tip to pleural dome, diversion and constriction C8; C7 riding on cervical rib tip, marked swelling C8	Yes	Surgical resection
2/35y/M	Ulnar distribution	M, S	right	2 years	Carpal tunnel syndrome on the right side and “trigger finger”	Stump rib C7, accessory ligament from tip to pleural dome, diversion and constriction C8, Th1; C7 riding on cervical rib tip, swelling C7, C8, Th1	Yes	Surgical resection
3/75y/M	Ulnar distribution	M, S	left	10 years	none	Stump rib C7, accessory ligament from the tip to the pleural dome, displacement and constriction of C8, riding of C7 on the rib	Yes	Conservative
4/26y/F	Median distribution	M, S	right	3 years	none	Staump rib C7, C6 root riding on edge tip, during head turn. Accessory ligament from the tip to the pleural dome	Yes	Conservative
5/33y/F	Median nerve territory, clinical diagnosis of carpal tunnel syndrome and ulnar neuropathy at the elbow	S	right	unknown	none	Stump rib on the right side with an accessory ligament to the pleural dome, compression, and displacement of C8, C7 riding on tip of the rib	Yes	Surgical resection
6/53y/F	Weakness of the left forearm, clumsiness of the hand, nuchal pain, paraesthesia in both hands, all fingers	M, S	left	10 years	none	Stump rib C7 with pseudarthrosis on the left, accessory ligament from tip to pleural dome, diversion and constriction C8	Yes	Surgical resection
7/56y/F	Diffuse pain radiating from the shoulder down the hand, territory not compatible with radicular or peripheral pattern	S	left	15 years	none	Stump rib on the left with riding and constriction of C8. Accessory ligament to the pleural dome.	Yes	Conservative
8/50y/F	Ulnar distribution, clinically suspected Loge de Guyon syndrome	M, S	bilateral	10 years	none	Stump rib C7 on both sides, only right side symptomatic, accessory ligament to the pleural dome, compression, and displacement of C8	Yes	Surgical resection
9/55y/F	Clinically atypical carpal tunnel syndrome on the right side	M, S	right	8 years	none	Stump rib C7 on the right side, accessory ligament to the pleural dome	Yes	Surgical resection
10/45y/F	Unclear dysesthesia of the hand, nuchal pain	M,S	right	3 years	none	Stump rib C7 on both sides. No direct contact to the nerves	Yes	Conservative
11/45y/M	Diffuse pain in the right hand	M	right	unknown	none	Stump rib C7 on the right side, accessory ligament to the pleural dome	Yes	Conservative
12/74y/F	C8 muscle weakness	M	right	unknown	none	Stump rib C7, accessory ligament from tip to pleural dome following medial border of scalenus medius muscle, indentation C8/Th1	No	Surgical resection
13/43y/F	Adductor pollicis brevis atrophy, dysesthesia C8/Th1	M,S	right	2 years	none	Stump rib C7, accessory ligament from tip to pleural dome following medial border of scalenus medius muscle, thickened inferior trunk C8/Th1	No	Surgical resection
14/54y/F	Atrophy of intrinsic hand muscles and abductor pollicis brevis muscle, pain C8/Th1, Raynaud syndrome	M,S	right	unknown	none	Stump rib C7, accessory ligament from tip to pleural dome following medial border of scalenus medius muscle, thickened inferior trunk C8/Th1	No	Surgical resection
15/25/F	Acute scapula alata with atrophy of the serratus anterior muscle. Hypesthesia of the thumb and forefinger.	M, S	right	one week	none	Stump rib C7 on both sides, accessory ligament from tip to pleural dome thickening and dispülacement C7/C8	Yes	Surgical resection
16/49y/F	Atrophy of intrinsic hand muscles and abductor pollicis brevis muscle, arm pain	M,S	right	15 y	none	Stump rib C7, accessory ligament from tip to pleural dome following medial border of scalenus medius muscle, thickened inferior trunk C8/Th1	No	Surgical resection

**FIGURE 3:**
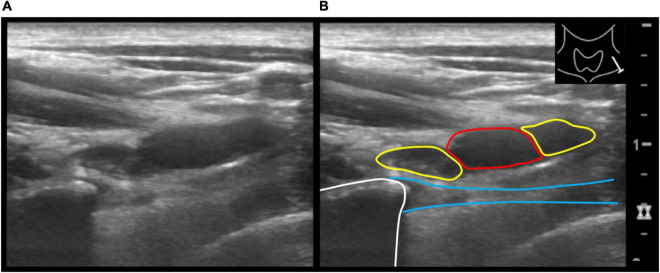
Axial ultrasound image of the supraclavicular neck region on the left showing the subclavian artery and anterior and posterior divisions of the brachial plexus **(A)**. Adjacent is the tip of the stump rib with partially depicted accessory ligament extending caudally from the tip of the stump. Illustration of the same ultrasound image **(B)**. Subclavian artery (red circle), the anterior and posterior divisions (yellow circles) the stump rib (white line) and the accessory ligament (blue lines).

**FIGURE 4:**
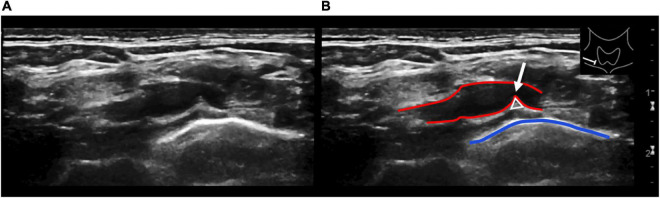
Longitudinal ultrasound view of the right subclavian artery at the level of the pleurocupular region **(A)**. Illustration of the same ultrasound image **(B)**. The red lines show the subclavian artery with indentation (white arrow) through the pleurocupular ligament (white triangle). The blue line identifies the pleural apex. Caution should be given to a depressed or angulated subclavian artery without associated flow reduction. This is a frequently seen and unspecific finding and therefore should not be overdiagnosed.

Sonographically, a common pattern of a slightly hyperechoic, anisotropic, linear structure that extended from the tip of a stump rib (*n* = 16) to the pleural cupula could be depicted (Figures 3, 4).

High-resolution ultrasound was able to assess the direct effect of the anatomical variants on the roots of the brachial plexus. In all cases, the ligament crossed the path of the subclavian artery, leading to a visible indentation of the artery and the C7 or C8 anterior ramus, which resulted in a significant course deviation of cervical roots even in the resting position. Five patients also showed an impression on the nerve fibers C8 and T1, accompanied by a focal thickening, compared to both the contralateral side and the neighboring nerve fibers.

**FIGURE 5:**
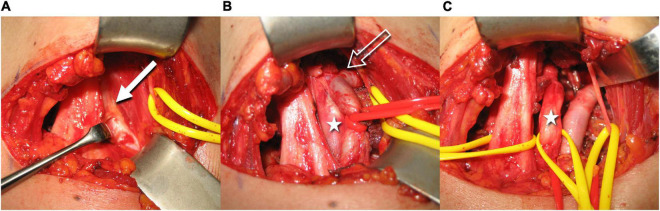
Intraoperative images illustrate the resection steps of the accessory ligament (the left side of the images is dorsal, top is cranial, right is ventral, and bottom is caudal). The white arrow shows the fibrous ligament above the neurovascular bundle **(A)**. After partial excision, a section of the brachial plexus is visible (white star). The constriction of the subclavian artery becomes evident (void white arrow) **(B)**. After complete removal of the fibrous band, the neurovascular bundle is now fully mobilized **(C)** (Courtesy of Prof. O. Aszmann).

Of all the eligible patients, two had already received surgical treatment for NTOS symptoms at the affected extremity without clinical benefit. A summary of clinical, neurological, and HRUS findings is given in Table 1. Eleven eligible patients subsequently received surgical treatment, where the HRUS finding of a costo- or transverso-cupular ligament was confirmed in all cases (Figure 5).

Twelve patients were examined with 3 Tesla MRI (Philips Achieva, Best, Netherlands). MRI showed (best seen in the parasagital STIR sequence) a thin, T2w-hypointense, band-like structure, extending caudally from the tip of the stump rib toward the pleural dome.

A clear T2w/STIR signal increase, a significant course alteration or a compression has been demonstrated in the inferior plexus portions (C7, C8, or T1 spinal nerves, inferior trunk, or medial cord). In all patients, MRI confirmed the findings of HRUS, the deviation, impingement, or swelling of the affected nerve roots, including the stump rib and the fibrous ligament.

**FIGURE 6:**
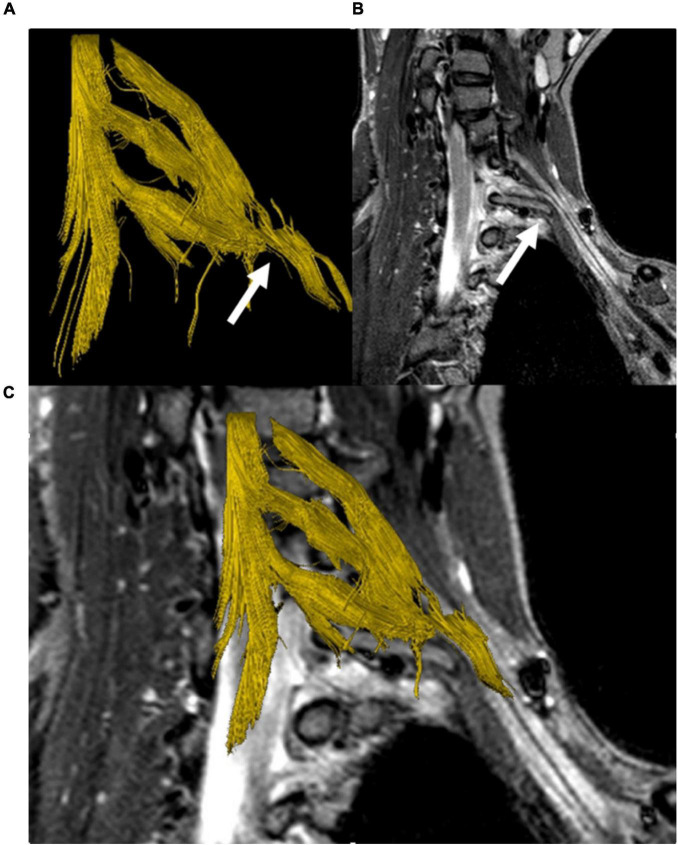
DTI-based fiber tractography of the left brachial plexus in a patient with a cervical stump rib (C7). 3D fiber tract reconstruction of neuronal fibers generated from DTI data **(A)**, parasagittal STIR-MRI sequence demonstrating the peak apex of the stump rib with clear elevation and a shift of the lower brachial plexus segments (white arrow) **(B)**. Merging of the fiber track and anatomical series **(C)** demonstrates the cervical rib’s impact on the course of neuronal fibers, causing a plausible, visible correlation between the MRI and DTI/FT data sets.

Diffusion tensor imaging-based fiber tractography of the brachial plexus and myelon was performed in ten patients. One data set could not be evaluated due to severe patient motion. In the remaining data sets, fibers of the upper brachial plexus nerve roots were visualized. Visualization of the inferior brachial plexus was limited due to breathing artifacts in 5/7 cases and did not reveal any abnormalities without knowledge of the structural MR findings. In 3/10 cases (Figure 6) a deviation of portions of the brachial plexus could be visualized in 3D by tractography.

## Discussion

This case series highlights the novel diagnostic feasibility of HRUS in patients with NTOS. We assessed 16 patients with NTOS where the HRUS diagnosis of accessory cervical ligaments that exert mechanical pressure on the brachial plexus were confirmed surgically or with MR tomography.

Accessory ligaments were first described by Emil Zuckerkandl at the end of the 19th century and have long been recognized in the anatomical and surgical literature as having a significant clinical relevance with regard to TOS and the thoracic outlet region (Roos, 1976, 1999; Redenbach and Nelems, 1998). Their occurrence is related to a hypertrophic transverse process, a cervical rib or stump rib, from which they strain toward the pleural cupula or the first rib–which corresponds to our findings in HRUS. In our series, HRUS was not only able to visualize the accessory cervical ligament, but also the cervical stump rib.

Although Doppler sonography is a well-established modality in the diagnosis of arterial TOS, the use of HRUS for the diagnosis of NTOS is still in its infancy. The patients in our sample showed indentation of the subclavian artery at a resting position and a moderate increase in the flow velocity during arm elevation; however, only one of the patients demonstrated a vessel stenosis considered to be hemodynamically relevant. The changing indentation of the vessel wall certainly represents significant chronic mechanical stress to the vessel and might well be a predisposing factor for future vessel damage. Nevertheless, as it has been described previously indentated or angulated subclavian artery without any hemodynamically relevant stenosis is non-specific and is seen also in patients with other conditions and should not be misinterpreted or overdiagnosed (Ferrante and Ferrante, 2017). The other typical imaging findings in patients our sample with NTOS generally did not vary from the HRUS findings in other compression neuropathies throughout the body and included thickening, indentation, or compression of nerve structures, as well as changes in echotexture with a loss of fascicular structure and increased hypoechogenicity, as well as course deviation. In our cohort, all of these imaging features were found to various degrees.

In addition to changes in neural structures and vessels, the results of our study indicate that HRUS may also be able to scan for bone and muscle variants or anomalies. All stump ribs, cervical ribs, or hypertrophied transverse processes were clearly depicted using HRUS. However, this needs to be targeted in future research.

We are aware of one study dealing with feasibility of high-field MRI in TOS (Baumer et al., 2014) who used a dedicated, customized surface coil and confirmed the possibility of visualization of fibrous bands compressing the brachial plexus (Baumer et al., 2014), findings that are also fully consistent with our MRI results. However, compared to MRI, HRUS brings several potential advantages: first, substantially higher spatial resolution using commercially available high-end equipment. Second, direct patient contact with the examiner, with the possibility of direct history taking and so-called “sono-palpation” (Boon et al., 2012), i.e., targeted application of pressure with the HRUS probe or the finger of the examiner and direct clinical correlation by a positive Tinel’s sign. Third, the possibility of a dynamic examination technique without the limitation of motion artifacts (breathing, pulsation of the vessels) (Vargas et al., 2009). This translates to several advantages, as TOS often shows a dynamic etiology and causes may be found both in detail or during arm or breathing motion.

Generally, we consider the approach of using HRUS as clinically oriented screening instrument and MRI as a complementary and confirmatory modality to be most appropriate for the assessment of patients with TOS. As both modalities need a certain level of experience, the examiner must be trained in peripheral nerve imaging. However, by combining both modalities patients would benefit from the high sensitivity of ultrasound and high specificity of MRI in assessing peripheral nerve compression by ligaments.

More advanced techniques such as DTI, are technically challenging, potentially limited by breathing motions in the plexus region in proximity of the lung apex. However, in some cases DTI based tractography allowed a 3D visualization of the nerve course deviation. This plastic and easily comprehensible visualization may be specifically useful for surgeons in the preparation of the decompressive surgery.

Due to the manual segmentation of DTI datasets currently required, deterministic tractography of the brachial plexus is still relatively time-consuming (20–30 min per plexus) and requires further technical improvements to become more applicable in clinical routine. In the future examiner independent probabilistic tractography approaches and optimized motion insensitive DTI sequence acquisition schemes will lead to more robust results.

All the above-mentioned factors may contribute to a quick and precise diagnosis in patients with NTOS. This is urgently needed, as these patients usually experience a long time until their diagnosis (a mean of 9 years in our sample) and the detection and often unspecific clinical presentation may lead to unnecessary surgery (Roos, 1999).

There are limitations to this study that arise from the retrospective case series design. A retrospective evaluation may be subject to a selection or measurement bias. The lack of a control group for the patients in this case series is another limitation. However, as patient outcomes or treatment success was not the subject of this study and the examinations were performed in a standardized fashion, all of these limitations can be considered less serious. This, together with the fact that the study sample is the largest sample that has been observed through imaging methods, makes this case series a valuable piece of information that is meant to be an entry point into further research on this topic.

In conclusion, this case series demonstrates that HRUS can directly visualize accessory costocupular ligaments in their anatomical context in patients with symptoms of NTOS. Combining HRUS with MR-Neurography is a highly sensitive and optimal imaging approach in these conditions and may, therefore, be able to significantly improve patient care for TOS patients.

## Data Availability Statement

Requests for access to the datasets used for this study can be directed to SJ, suren.jengojan@meduniwien.ac.at and MB, maria.bernathova@meduniwien.ac.at.

## Ethics Statement

The studies involving human participants were reviewed and approved by the Ethics Committee of the Medical University of Vienna. Written informed consent for participation was not required for this study in accordance with the national legislation and the institutional requirements.

## Author Contributions

GB, SJ, GK, MB, and TM contributed to conception and design of the study. MB and PS organized the database. PS performed the statistical analysis. MB and TM wrote the first draft of the manuscript. GB, SJ, and GK wrote sections of the manuscript. SJ wrote the submitted version. All authors contributed to manuscript revision, read, and approved the submitted version.

## Conflict of Interest

The authors declare that the research was conducted in the absence of any commercial or financial relationships that could be construed as a potential conflict of interest.

## Publisher’s Note

All claims expressed in this article are solely those of the authors and do not necessarily represent those of their affiliated organizations, or those of the publisher, the editors and the reviewers. Any product that may be evaluated in this article, or claim that may be made by its manufacturer, is not guaranteed or endorsed by the publisher.
